# Impact of Noise and Background on Measurement Uncertainties in Luminescence Thermometry

**DOI:** 10.1021/acsphotonics.2c00039

**Published:** 2022-03-11

**Authors:** Thomas P. van Swieten, Andries Meijerink, Freddy T. Rabouw

**Affiliations:** Debye Institute for Nanomaterials Science, Utrecht University, 3584 CC Utrecht, The Netherlands

**Keywords:** luminescence thermometry, temperature uncertainty, statistics, (EM)CCD, background, absorption cross section

## Abstract

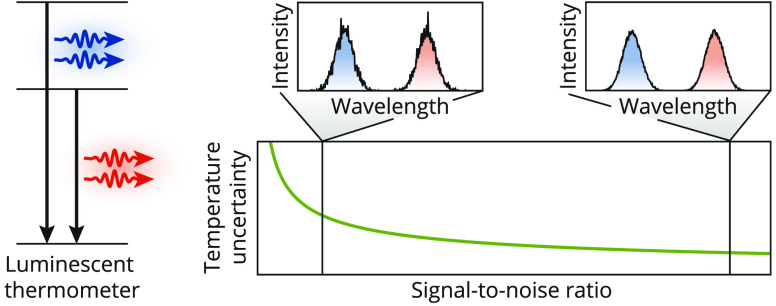

Materials with temperature-dependent luminescence can be used as local thermometers when incorporated in, for example, a biological environment or chemical reactor. Researchers have continuously developed new materials aiming for the highest sensitivity of luminescence to temperature. Although the comparison of luminescent materials based on their temperature sensitivity is convenient, this parameter gives an incomplete description of the potential performance of the materials in applications. Here, we demonstrate how the precision of a temperature measurement with luminescent nanocrystals depends not only on the temperature sensitivity of the nanocrystals but also on their luminescence strength compared to measurement noise and background signal. After first determining the noise characteristics of our instrumentation, we show how the uncertainty of a temperature measurement can be predicted quantitatively. Our predictions match the temperature uncertainties that we extract from repeated measurements, over a wide temperature range (303–473 K), for different CCD readout settings, and for different background levels. The work presented here is the first study that incorporates all of these practical issues to accurately calculate the uncertainty of luminescent nanothermometers. This method will be important for the optimization and development of luminescent nanothermometers.

Nanomaterials with temperature-dependent luminescence are one of the most versatile thermometers on the microscopic scale with applications in biology, electronics, and catalysis.^1−3^ The temperature of a nanothermometer is determined by recording its emission spectrum or its luminescence lifetime. The intensity ratio between two emission bands is most frequently considered because this parameter is insensitive to fluctuations in the excitation intensity, changes in alignment, and scattering of the luminescence. The relative sensitivity, *S*_r_ (in % per K), expresses how strongly the intensity ratio changes with temperature and is thus a measure for the measurement accuracy. *S*_r_ is an intrinsic property of a thermometer material (which however depends on temperature) and is easily determined by measuring emission spectra over a range of temperatures. Newly developed thermometer materials are therefore often characterized and compared in terms of this parameter.^4,5^

In practice, the reliability of temperature readout depends not only on the relative sensitivity but also on the signal-to-noise ratio of a measurement. These parameters together determine the temperature uncertainty, σ_*T*_. Current methods to determine σ_*T*_ are diverse. The most direct method is experimentally recording a series of luminescence spectra and calculating the standard deviation of the extracted temperatures.^6^ Alternatively, the noise level on a single spectrum may be estimated from fluctuations in the baseline. The latter method underestimates the temperature uncertainty because it fails to take the noise on detected photons into account.^7^ More importantly, both methods are often used in idealized circumstances where background signal is minimal, a large amount of thermometer material is measured, luminescence is efficiently collected, and/or long measurement times are used. The extracted values of σ_*T*_ depend strongly on these circumstances. In contrast to *S*_r_, σ_*T*_ is not an intrinsic property of a (nano)thermometer material. Consequently, user-to-user differences have caused variations in reported uncertainties of several orders of magnitude for the same thermometer, while some measurement conditions such as the environment of the thermometer were similar.^4^ It is not clear to what extent these reported values of σ_*T*_ are relevant for actual applications of the (nano)thermometers, which may put restrictions on the measurement procedure and/or introduce background fluorescence and blackbody radiation.^8^ In addition, undesired emissions from the thermometer itself, for example, from higher-excited levels, can interfere with temperature measurements.^9^ Although subtracting a reference spectrum of any background signal removes the systematic error,^10^ the influence on the temperature uncertainty remains. It is currently unclear how these practical complications affect the performance of luminescent (nano)thermometers. This makes a fair comparison of potential thermometer materials impossible.

In this article, we use the statistics of photon detection to quantify how noise and background signal affect the temperature uncertainty of a luminescence (nano)thermometry experiment. Not only the properties of the thermometer material are important but also the characteristics of the detector and the sample. We first measure the background-free upconversion luminescence of NaYF_4_:Er^3+^(2%),Yb^3+^(18%) nanocrystals using a conventional CCD and characterize the different types of detector noise. Using error propagation, we quantitatively explain the temperature uncertainties determined by recording a series of spectra, which increases with the set temperature. We study the impact of detector noise by recording upconversion luminescence of nanocrystals with electron multiplication gain using an electron-multiplying CCD (EMCCD). An increase in gain boosts the signal to overcome readout noise and thus reduces the temperature uncertainty. Finally, we examine the effect of background signal. Even if we subtract the background signal, the experimental temperature uncertainty increases with higher background levels as predicted from the larger error on the number of detected photons. These new methods to calculate the uncertainty show that not only the relative sensitivity *S*_r_ of a thermometer determines its performance but also the achievable signal-to-noise ratio. The temperature uncertainty σ_*T*_ depends strongly on measurement conditions and is therefore a poor parameter to compare the potential of thermometer materials. We propose alternative metrics that could be considered.

## Results

**Figure 1:**
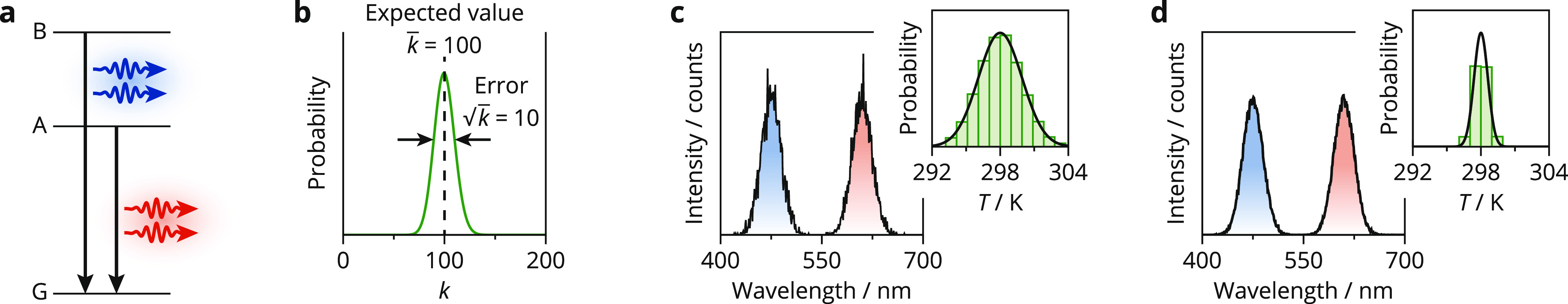
Temperature uncertainty achieved with a model thermometer. (a) Energy level diagram of the model thermometer, in which the solid black arrows represent the radiative decay pathways. (b) Poisson distribution with mean *k̅* = 100 and standard deviation √*k̅* = 10. (c) Simulated luminescence spectrum comprising two Gaussian emission bands with Poissonian detection noise. The inset shows a histogram of the temperatures that are extracted from 10 000 simulated spectra using the ratio of integrated counts, a physical temperature of 298 K, and a relative sensitivity of 1% K^–1^. The black line is a normal distribution with a mean of 298 K and a standard deviation that is calculated via eq 3. (d) Same as in (c) but for a total luminescence intensity that is 10 times higher.

We first discuss the uncertainty achieved with a model thermometer based on two emissive excited levels A and B (Figure 1a). An increase in temperature affects the relative intensities emitted by these levels, resulting in a change in the intensity ratio in the emission spectrum. In a typical experiment, these emissions are spectrally separated by a grating and captured by a CCD, photomultiplier tube, or photodiode array. The photosensitive material in the detector converts the incident photons to photoelectrons. The number of photoelectrons *k* recorded in one exposure will follow the Poisson distribution
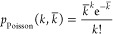
1where *k̅* is the expected number of photoelectrons, which is proportional to the product of the photon flux and the acquisition time and is in general different for levels A and B. An interesting property of the Poisson distribution is that the standard deviation is equal to the square root of the expected value (Figure 1b). The next step in the detection process is the translation of photoelectrons to digital counts for each pixel, which enables the construction of the emission spectrum. For a luminescence thermometry experiment, the observables of interest in the spectrum are the integrated counts of the emissions from levels A and B: *n*_A_ and *n*_B_, respectively. As *n*_A_ and *n*_B_ are independent random variables, the measurement error on the intensity ratio *R* = *n*_B_/*n*_A_ follows from error propagation^11^
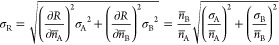
2Here, *n̅*_A,B_ are the expected counts of A and B with corresponding variances σ_A,B_^2^ and *n̅*_A,B_/σ_A,B_ are the signal-to-noise ratios on *n*_A_ and *n*_B_. Assuming that the errors and expected counts are related as described by the Poisson distribution, we expect lower σ_*R*_ for higher counts.

Conversion of *R* to a temperature value requires knowledge of the relative sensitivity. This is often obtained by calibrating the spectral response of the thermometer over a range of temperatures. Any error in the calibrated relative sensitivity leads to a systematic difference between the measured and physical temperature. However, the random error σ_*T*_ on the measured temperature only depends on the probability distribution function of the measured *R* and on the relative sensitivity of the thermometer^11^
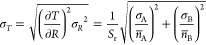
3This shows that, as expected, the temperature uncertainty decreases with the increasing signal-to-noise ratio. An alternative approach of luminescence thermometry relies on the shift of an emission band at varying temperatures. In the Supporting Information, we use a similar analysis as above to determine the temperature uncertainty of measurements based on a spectral shift. Finally, we verify eq 3 by simulating luminescence spectra with two emissions bands and determine the temperature from the ratio of the simulated counts (Figure 1c). These simulated temperatures follow a normal distribution with a standard deviation that matches σ_*T*_ calculated using eq 3. The distribution of temperatures would deviate from normal if *n̅*_A,B_ becomes of order unity, rather than ≫ 1 as we consider in Figure 1 and is typical in experiments. An increase in counts results in a narrower distribution of measured temperatures, consistent with eq 3 (Figure 1d). We thus understand quantitatively how experiments with higher counts, performed with, for example, longer acquisition times or brighter thermometers, have a lower temperature uncertainty.

**Figure 2:**
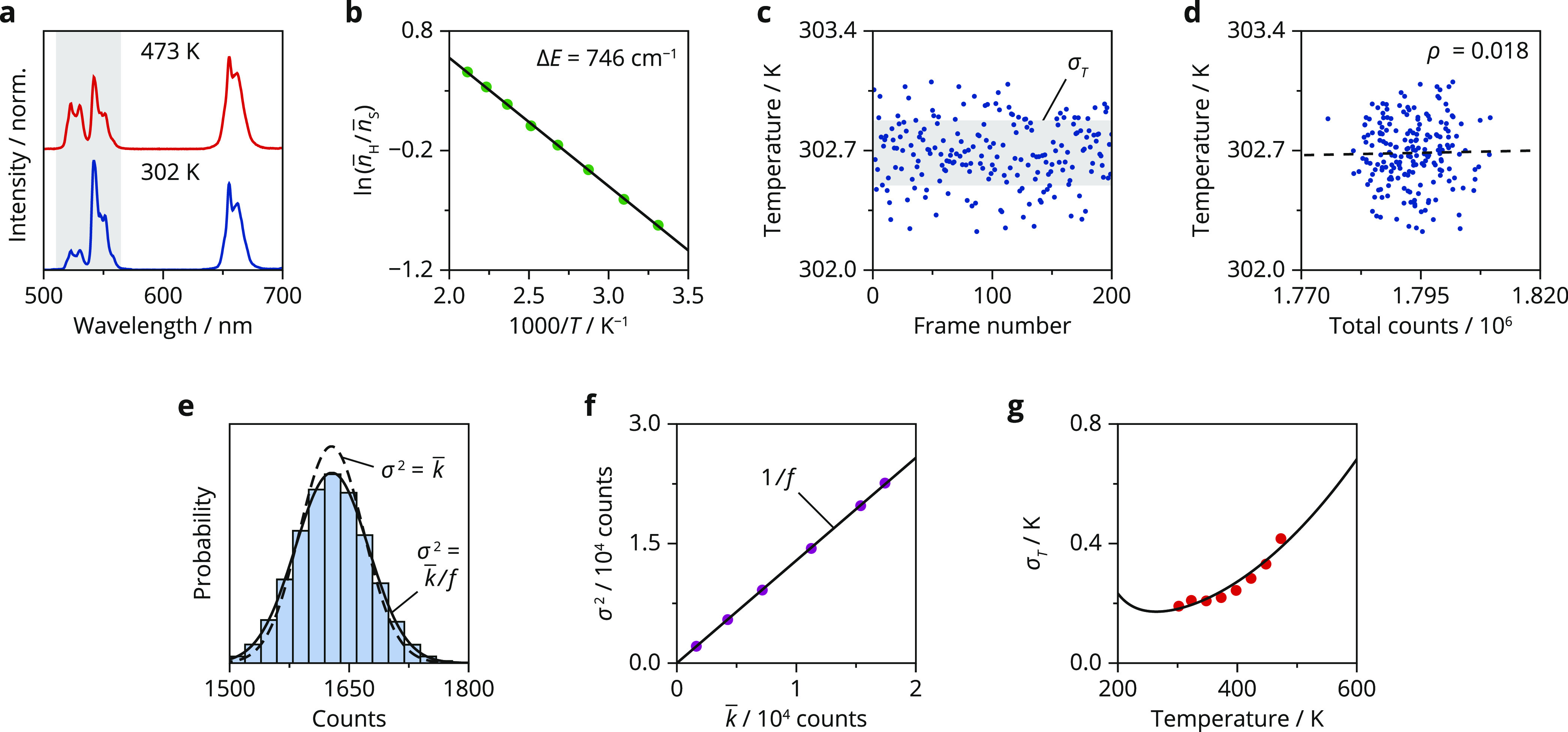
Experimental temperature uncertainty. (a) Upconversion luminescence of dried NaYF_4_:Er^3+^(2%),Yb^3+^(18%) nanocrystals upon 980 nm excitation at 302 K (blue) and 473 K (red). (b) Logarithm of the ratio between the ^2^H_11/2_ and ^4^S_3/2_ emission with integration ranges 516–534 and 538–545 nm (green dots), respectively. The black line is a fit of the experimental ratios to Boltzmann’s distribution (eq 4), yielding values of 746 cm^–1^ for Δ*E* and 15.2 for *C*. (c) Temperatures extracted from 200 experimental spectra using the calibration in (b). The thermocouple in our heating stage measured a temperature of 302 K during the acquisition of spectra, showing a small deviation with the mean of the temperatures extracted from the spectra, likely caused by a systematic error in the calibration. The gray shaded area covers the temperature range of the mean ± standard deviation. (d) Correlation between the measured temperature and the total counts within the integration ranges of the ^2^H_11/2_ and ^4^S_3/2_ emissions. The correlation coefficient (ρ) is close to zero, indicating that measured temperature and total counts are uncorrelated. (e) Distribution of counts per 1000 ms frame, for pixels on our CCD camera showing an average of 1629 counts/1000 ms when measured over 200 frames. The camera recorded the reflection of a white lamp on a microscopy slide. The solid line is a fit of the experimental data to the normal distribution (*k̅* = 1629, σ^2^ = 2112), and the dashed line shows the Poisson distribution with *k̅* = 1629. (f) Plot of the variance against the mean (purple dots) measured via the procedure in (e) for different intensities of the white lamp. The black line is a fit of the experimental data to the model σ^2^ = *k̅*/*f* + σ_r_^2^, where *f* = 0.78 is the analog-to-digital conversion factor and σ_r_^2^ = 57 is the readout variance of one pixel. (g) Temperature uncertainties at various physical temperatures obtained via the procedure in (c) (red dots). The black line is the temperature uncertainty calculated using eq 3, with no fit parameters.

In Figure 2, we experimentally study the temperature uncertainty of thermometry measurements at elevated temperatures. We acquired spectra with a CCD camera because this is the most frequently used detector in the luminescence thermometry community. The CCD camera conveniently records an entire spectrum within one capture. In contrast, step-wise acquisition of a spectrum with a scanning monochromator and single-point detector such as a photomultiplier tube leads to additional temperature errors if the excitation intensity fluctuates during the measurement. We use NaYF_4_:Er^3+^(2%),Yb^3+^(18%) nanocrystals as thermometers because green upconversion emission from this popular thermometer material can be excited with 980 nm light, preventing background fluorescence.^12^Figure 2a shows the luminescence spectra, in which the two emission bands at 540 and 520 nm are due to radiative decay from the thermally coupled levels in Er^3+^, ^4^S_3/2_, and ^2^H_11/2_, respectively. An increase in temperature (*T*) changes the ratio of the expected counts within the emission bands of ^4^S_3/2_ and ^2^H_11/2_, *n̅*_S_ and *n̅*_H_, respectively, following Boltzmann’s distribution
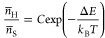
4where Δ*E* is the energy gap between these levels, *k*_B_ is Boltzmann’s constant, and *C* is the pre-exponential factor that includes the degeneracies and radiative decay rates from the two levels to the ground state. To use this relation as a calibration of our thermometer, we average 200 spectra and obtain *n̅*_S_ and *n̅*_H_ by summing the counts of all pixels within the integration boundaries of the corresponding emission bands. We then fit the ratio measured at various temperatures to eq 4 and find a value of 746 cm^–1^ for Δ*E* and 15.2 for *C* (Figure 2b).^13^ We use this calibration to convert the measured intensity ratios from spectra with different levels of noise to apparent temperatures.

Figure 2c shows the temperatures that we extracted from a series of spectra using the calibration of Figure 2b.^14^ These values are evenly distributed around the mean, which is a sign of a stable physical temperature during the measurement. Correlations between the extracted temperature and the total green luminescence counts could indicate that the laser heats the sample as variations in laser intensity would result in higher count rates coinciding with more laser heating. Experiments at higher laser powers do show such correlations (Figure S2), which indicates that increasing the excitation intensity to reduce the uncertainty can induce a systematic error on temperature readout. This is not observed in our experiments shown in Figure 2d. We therefore used the standard deviation of the measured temperatures as the experimental temperature uncertainty at a fixed sample temperature. Fluctuations in excitation intensity below the heating threshold do not affect the intensity ratio nor the signal-to-noise ratio (Figure S3).

To understand the magnitude of the variations in measured temperature (Figure 2c), we must consider the noise generated by our detector. The main noise sources in a CCD measurement are counting noise due to the statistics of incident photons and readout noise due to the translation of photoelectrons to digital counts by the analog-to-digital converter (ADC).^15^ We characterize these by acquiring a large set of 200 reference images on our CCD camera, illuminating it with a white lamp, and histogramming the digital counts of pixels with the same mean (Figure 2e). The distribution of digital counts approximates a normal distribution with a variance that is, in our case, slightly larger than the corresponding Poisson distribution would have, taking the single-pixel readout variance into account. This difference is due to the conversion of photoelectrons to digital counts, which changes the variance on the output counts by the ADC factor, *f*.^16,17^Figure 2f shows a fit of the experimental variances to a model that includes the ADC factor and the readout noise
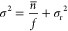
5We find a value of 0.78 for the ADC factor, which is specific to our camera in the used settings. If an emission band is integrated over *N* camera pixels, the variance of the total readout noise on this band is *N* times larger than the single-pixel readout variance σ_r_^2^ = 57. We can now insert this expression and the relative sensitivity of a Boltzmann thermometer, Δ*E*/*k*_B_*T*^2^,^18^ into eq 3 to calculate the expected temperature uncertainty from a luminescence spectrum.

Figure 2g shows the temperature dependence of the uncertainty by comparing experiments with the theoretical trend (eqs 3–5). Using the method presented in Figure 2c, we determine the temperature uncertainty at various physical temperatures and find values of 0.2 K at room temperature increasing to more than 0.4 K at 473 K.^19,20^ Between the different physical temperatures of the experiment, the total counts within the spectrum varied slightly—a decrease is likely due to thermal quenching and an increase could indicate water desorption from the surface of the dried nanocrystals.^21^ This affects the signal-to-noise ratio and thus obscures the impact of the intensity ratio on the temperature uncertainty. We therefore kept the sum of *n̅*_S_ and *n̅*_H_ roughly constant at 2 × 10^6^ counts, which allows us to separately calculate *n̅*_H_ and *n̅*_S_ using eq 4 in a range of physical temperatures. We can therefore use eq 4 to calculate *n̅*_H_ and *n̅*_S_ separately, depending on the physical temperature. Inserting these values, along with the detector characteristics (eq 5), into eq 3 yields the theoretical uncertainty (black line in Figure 2g). The calculated uncertainties agree well with the experimental values, without any fit parameters. We therefore conclude that after proper characterization of the photodetector, error propagation correctly predicts the experimental uncertainty and its temperature dependence.

**Figure 3:**
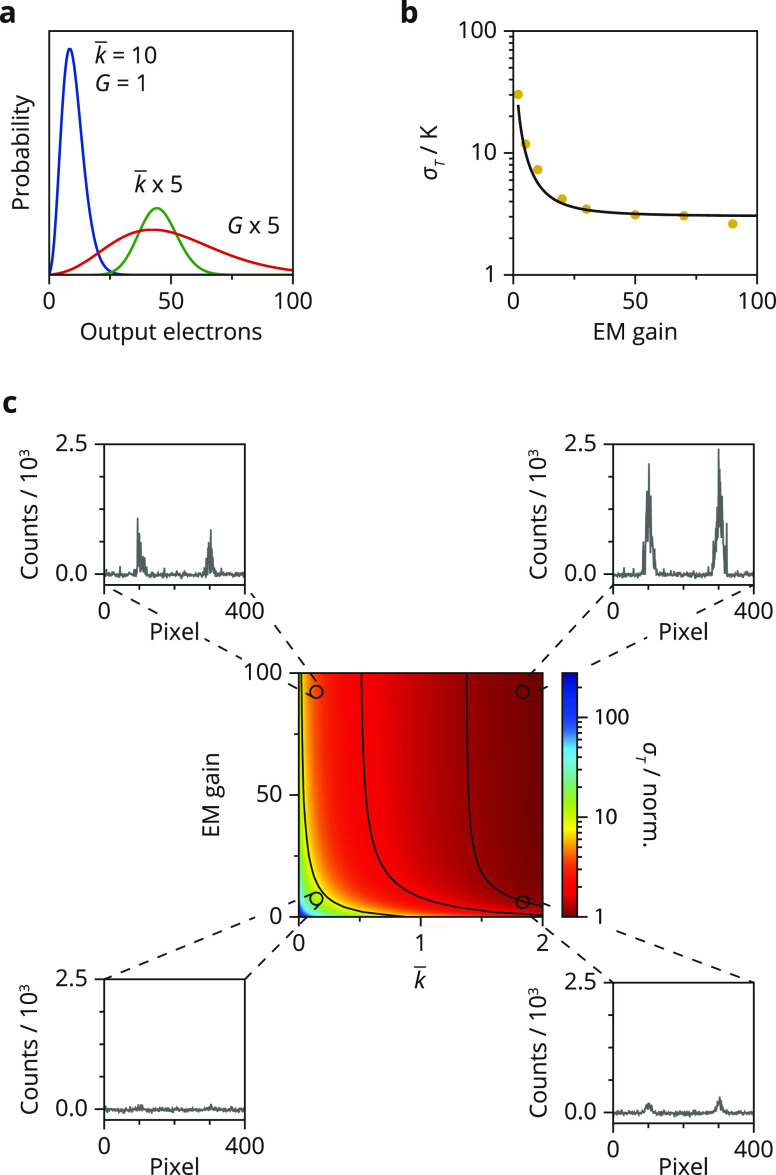
Effect of electron multiplication on temperature uncertainty. (a) Distribution of output electrons for different input photoelectrons *k̅* and EM gains *G*. This distribution is obtained by convolution of the gamma distribution and the Poisson distribution. (b) Experimental temperature uncertainties obtained from 200 upconversion spectra for various levels of EM gain ranging from 2 to 90 (yellow dots). The average numbers of photoelectrons generated by a pixel are 5 and 30 for the ^2^H_11/2_ and ^4^S_3/2_ emissions, respectively. The solid black line is the temperature uncertainty calculated via eqs 3 and 7, with no fit parameters. (c) Color map of the temperature uncertainty as a function of the expected photoelectrons *k̅* per pixel and the EM gain. The temperature uncertainties in the color map were calculated via the expected value of the output electrons, excluding spurious electrons, and the variance of all output electrons (eq S1). All uncertainties were normalized to the minimum value within the map. The contour lines correspond to σ_*T*_ values of 1.2, 2, and 8 K. We obtain the simulated spectra in (c) by drawing random numbers of output electrons from the distribution of eq S1, with as an input a spectrum consisting of two peaks with Gaussian shape of equal amplitude covering a total of 400 pixels. The four insets show example spectra simulated for the experimental settings to which they are linked in the color map. We set the readout noise for each pixel to σ_r_ = 26, matching that of our EMCCD detector at a 30 MHz readout rate and preamplifier gain 2. We assume a probability of spurious charges of *p*_s_ = 0.0004 to give the simulated spectra the characteristic background noise of electron multiplication.

As a further illustration of the effect of the detector noise characteristics on a temperature measurement, we consider the effect of electron multiplication (EM) in an EMCCD. Emerging applications of luminescent nanomaterials, such as single-particle thermometry, require photodetectors that are able to record extremely weak signals.^22,23^ EMCCDs could offer a solution as they enhance the signal by orders of magnitude, compared to conventional CCDs, but the electron multiplication process causes additional noise.^15,16^ We start by considering the detection of photons and generation of photoelectrons, which in both a conventional CCD and an EMCCD follows Poisson statistics. Both types of cameras then transfer the photoelectrons to the ADC via the serial readout register and convert them to digital counts. In an EMCCD camera, the readout register is extended with additional registers that, depending on the applied voltage, multiply the number of photoelectrons and thus boost the signal. In practice, the output electrons pass through hundreds of multiplication registers, resulting in a total EM gain *G*. The number of output electrons *n*_out_ follows the gamma distribution
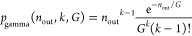
6Here, *k* is the number of photoelectrons generated by a CCD pixel, which enter the multiplication registers. Figure 3a shows the probability distribution of *n*_out_ as a function of the expected number of input photoelectrons *k̅* and the EM gain *G*. EM produces an expected number of counts of *n̅*_out_ = *k̅G* with a variance that approximates σ_*n*_^2^ = 2*k̅G*^2^/*f* (eq S1 and Figure S4). The signal-to-EM-counting-noise ratio , where readout noise is excluded, is thus independent of the gain factor. This derivation shows that EMCCD measurements have an additional counting noise of √2, commonly referred to as the excess noise factor. Therefore, EM gain can only improve a temperature measurement if *n*_out_ is small with respect to other noise sources.

In Figure 3b, we compare the experimental temperature uncertainty at various levels of EM gain with theoretical predictions. First, we acquired 200 experimental spectra with an EM gain of only a factor 2 while keeping the number of incident photons per pixel low. This resulted in an extremely high uncertainty of 30 K. Increasing the EM gain to values of 25 causes a sharp decline of the uncertainty to 3 K. The effect of even higher EM gains is weak. We again explain this trend using eqs 3 and 5 (solid line) by realizing that the variance of the ^4^S_3/2_ and ^2^H_11/2_ counts is due to a combination Poissonian counting noise amplified by the ADC factor *f* and the EM gain factor *G*^2^ and readout noise. [For photon fluxes relevant for luminescence thermometry, we can neglect noise due to spurious electrons created during shifting of charges through the multiplication register (Figure S5).^15,16^] EM gain increases the signal (as well as the counting noise) with respect to the readout noise
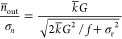
7Figure 3c illustrates the effect of EM gain for a range of expected photoelectrons. We observe the highest uncertainty in the bottom left of the map, where both the EM gain and the number of expected photoelectrons are low—the corresponding simulated spectrum barely shows emission. For all numbers of expected photoelectrons, we observe a rapid decrease of the uncertainty with increasing gain, although this effect becomes negligible once readout noise is overcome (Figure S6). Indeed, the signal-to-total-noise ratio (eq 7) approaches a constant value of  for large *G* and EM cannot improve it further (Figure S7). In practice, EM gain is thus useful if and only if the signal is weak compared to readout noise.

**Figure 4:**
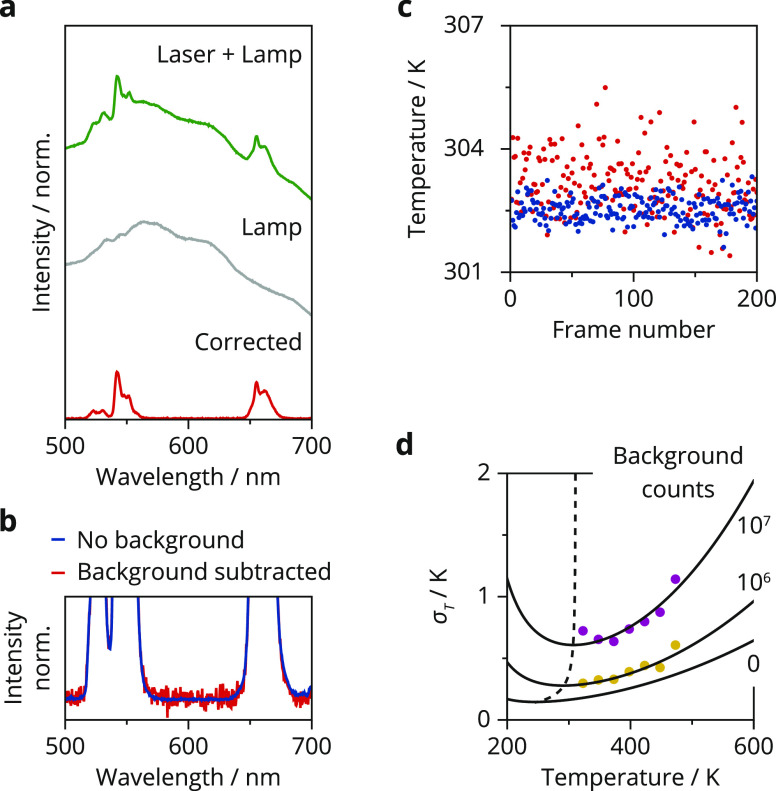
Influence of background subtraction on temperature uncertainty. (a) Reflection spectrum of the white lamp illuminating dried NaYF_4_:Er^3+^(2%), Yb^3+^(18%) nanocrystals without (gray) and with simultaneous 980 nm excitation (green). Subtraction of the gray spectrum from the green spectrum yields the corrected upconversion spectrum (red). The total signal (thermometer) and background (lamp) counts within the integration ranges of the ^2^H_11/2_ and ^4^S_3/2_ emission are 2 × 10^6^ and 2 × 10^7^, respectively. (b) Zoom-in on the noise of a spectrum acquired without background (blue) and with a broadband background that is subsequently subtracted (red). (c) Calibration curve of Figure 2b was used to convert a series of 200 spectra with no background (blue) and with lamp background subtracted (red) to temperatures. The red and blue dots show a systematic difference of the mean, which we attribute to an error introduced by recording and subtracting the reference spectrum, for example, because the lamp spectrum fluctuated slightly over time. (d) Temperature uncertainties as a function of set temperature, measured by comparing the apparent temperature of 200 background-subtracted spectra. The solid black lines are the expected temperature uncertainties for 2 × 10^6^ (yellow dots) and 2 × 10^7^ (purple dots) subtracted background counts, calculated using eq 4. The expected background counts on the ^2^H_11/2_ and ^4^S_3/2_ emissions are obtained by taking the sum of the counts in the subtracted spectrum between 516–534 and 538–545 nm, respectively, averaged over 200 spectra. The dashed black line marks the minimum of the temperature uncertainty for all subtracted background counts.

Besides the photodetector, background emission by the surroundings of the thermometer can be another source of uncertainty, which is relevant when the thermometer is used in realistic experimental conditions.^24^ We discuss how such a distortion of the spectrum affects the temperature uncertainty even after subtraction of the background. Figure 4a shows how we have mimicked this experimental issue: we have measured the upconversion luminescence with and without an additional broadband background signal from a white lamp. Subtracting a reference measurement of the lamp recovers a clean thermometer spectrum from the experiment with background. However, the noise on the background signal cannot be removed. The corrected spectrum therefore contains more noise compared to the background-free upconversion emission spectrum (Figure 4b). This translates to an increased temperature uncertainty (Figure 4c). We can further understand this from the expression for the variance σ_b,*i*_^2^ in the counts from emitting state *i* after background removal
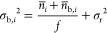
8where *n̅*_b,*i*_ is the expected number of background counts removed. Additional counts from dark current in the photodectector have an equivalent impact on the temperature uncertainty as background emissions. Again, inserting this expression into eq 3 gives the theoretical temperature uncertainty after background removal.

The measured uncertainties as a function of physical temperature match the predicted values for a range of different background levels (Figure 4d). We observe higher absolute values of the uncertainty with an increasing background. This effect is as large as a factor of 3 for the background level in Figure 4a, even though we could subtract the background using a reference measurement. We further observe that the minimum of the temperature uncertainty (dashed line) shifts to higher set temperatures with increasing background counts. In practice, low levels of background are challenging to completely avoid, especially at elevated temperatures where blackbody radiation becomes an issue. Figure 4 shows how this affects the absolute value of temperature uncertainty as well as the optimal operating temperature of a thermometer compared to idealized measurement conditions without background signal.

Our work clearly demonstrates how the precision of a temperature measurement depends not only on the relative sensitivity of the thermometer but also on the measurement conditions. As these measurement conditions will be different for different applications, this raises the question of how to define a relevant metric to compare thermometers. Currently, the achieved temperature uncertainty is frequently reported in the literature. However, our results show that the temperature uncertainty is *not* a fundamental property of the thermometer. Temperature uncertainties measured under idealized experimental conditions are difficult to compare and may not be relevant for applications. In particular, different experimental settings can yield wildly different contributions of various noise sources. However, whatever the specific experimental noise contributions are, the temperature uncertainty is always minimal for a high relative sensitivity *S*_r_ and for a strong luminescence signal (eq 3). Although *S*_r_ of newly developed thermometer materials is commonly reported,^26^ the potential signal strength is hardly considered.

The realization that signal strength is essential for precise temperature measurement makes it possible to identify relevant parameters, in addition to *S*_r_, that define a good thermometer. The expected counts on emissions A and B can be written as
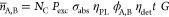
9Here, absorption of excitation light is determined by the number of luminescent centers in the excitation volume (*N*_C_) and the absorption cross section σ_abs_ per luminescent center. The luminescence further scales with the photoluminescence quantum yield η_PL_ of the relevant thermometer emission lines and the temperature-dependent populations that define the fractions ϕ_A,B_ of emission coming from A or B. The spectroscopic equipment sets the detection efficiency η_det_. The integration time *t*, the excitation power used *P*_exc_, and the EM gain *G* can be chosen by the experimentalist.

In eq 9, we can distinguish the experimental factors (η_det_, *P*_exc_, *t*, *N*_C_, and *G*) from the thermometer properties (σ_abs_, η_PL_, and ϕ_A,B_). The experimental factors will depend on the available equipment and the type of sample. The freedom to choose a long *t*, high *N*_C_, or high *P*_exc_ may be restricted if the sample is not static, if the sample volume is small, or if strong excitation induces laser heating (which also depends on heat dissipation in the sample). The values of these parameters are not intrinsic thermometer properties but depend strongly on the application. Another factor that affects the temperature uncertainty is the emission wavelength of the thermometer because it determines the required type of detector and therefore the amount of dark current. Infrared detectors typically have a high dark current due to the small band gap of the photosensitive material, resulting in a relatively high uncertainty for infrared-emitting thermometers. Equation 9 also contains some intrinsic properties that can vary by orders of magnitude between different materials. We propose that a fair comparison between potential thermometer materials should consider these intrinsic parameters. For practical applications, high σ_abs_ and η_PL_ for a given doping content *N*_C_ and excitation power *P*_exc_ are as important as a high thermal sensitivity of ϕ_A,B_.^27^ These intrinsic properties of the thermometer determine the achievable signal compared to various application-related issues, like background fluorescence or blackbody radiation.

Studies of new thermometers often include measurements of the sensitivity, but experimental values of η_PL_ and σ_abs_ are rare. Depending on the doping concentration, the synthesis procedure, and the excitation power, η_PL_ can vary over a few orders of magnitude, and it is therefore an important parameter to report. A well-established method to measure η_PL_ is to determine the number of absorbed and emitted photons of a sample using an integrating sphere. This has already improved the design and synthesis of thermometer materials. For example, recent studies on NaYF_4_:Er^3+^,Yb^3+^ nanocrystals have optimized the quantum yield of NaYF_4_:Er^3+^,Yb^3+^ in a range of excitation powers, reaching values comparable to bulk material.^28^

**Figure 5:**
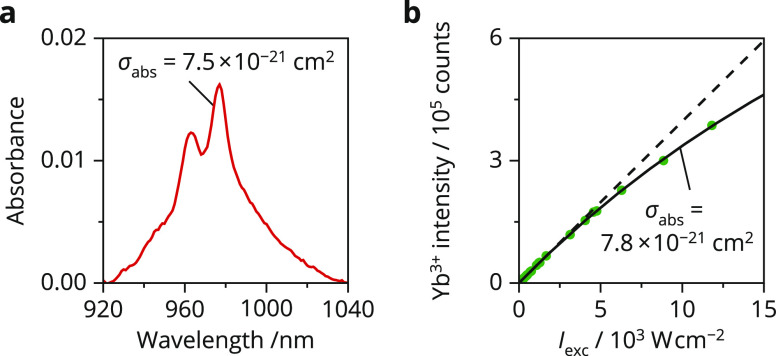
Experimental methods to determine the absorption cross section. (a) Absorption spectrum of NaYF_4_:Yb^3+^(18%) nanocrystals dispersed in cyclohexane. The spectrum shows a clear absorption band due to the ^2^F_7/2_ → ^2^F_5/2_ transition of Yb^3+^. Rayleigh scattering caused a background of roughly 0.0015 absorbance units, which was subtracted from the spectrum. The concentration of the bare nanocrystals without ligands is 10 mg/mL, which corresponds to a concentration of Yb^3+^ ions of 5.4 × 10^18^ cm^–3^. Taking the inhomogeneous dielectric surroundings of the Yb^3+^ ions inside the dispersed nanocrystal into account,^25^ this translates into an absorption cross section of σ_abs_ = 7.5 × 10^–21^ cm^2^ at 977 nm. (b) Intensity of the Yb^3+^ luminescence measured on microcrystalline NaYF_4_:Yb^3+^(18%) for various excitation intensities *I*_exc_ of 980 nm light (green dots). The solid black line is a fit of the experimental data to eq 10, which yields a σ_abs_ value of 7.8 × 10^–21^ cm^2^ at 980 nm. The dashed line is a linear fit to the low-excitation-intensity data to clearly visualize the nonlinear trend of the high-excitation-intensity data.

Characterizing σ_abs_ can be more challenging, especially for microcrystalline samples where strong light scattering prevents measuring optical absorption over a well-defined path length. We study a clear dispersion of Yb^3+^-doped NaYF_4_ nanocrystals with absorption spectroscopy to show that for a specific ion–host combination σ_abs_ simply follows Lambert–Beer’s law (Figure 5a).^29−31^ We find a maximum value of 7.5 × 10^–21^ cm^2^ at 977 nm, which matches literature values obtained from absorption measurements of single crystals^32,33^ and from the kinetics of upconversion luminescence.^34^ These values are an order of magnitude lower than the value that Sui et al. obtain from Judd–Ofelt parameters and an experimental photoluminescence decay rate.^35,36^ Nonradiative processes, indicated by the multiexponential decay curve, have likely resulted in an overestimation of the spontaneous emission rate and, thus, of the absorption cross section. Another method has recently been developed by our group, which extracts σ_abs_ from the luminescence saturation characteristics.^37,38^ In contrast to absorption measurements, it works well on microcrystalline samples and is thus a suitable alternative for samples that cannot be synthesized in nanocrystalline form. This method requires standard spectroscopic equipment, a continuous-wave laser, a lens to achieve sufficiently high excitation intensities, and careful characterization of the excitation spot on the sample (Figure S8). We used this method to acquire the luminescence of Yb^3+^-doped NaYF_4_ microcrystals at various excitation intensities (*I*_exc_), revealing clear signs of saturation above 5 kW cm^–2^ (Figure 5b).^39^ We fit this trend to the steady-state emission intensity of an excited two-level system that suffers from ground-state depletion.
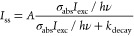
10where *A* is a scaling constant, *h*ν is the energy of an excitation photon, and *k*_decay_ is the total decay rate of the excited ion.^40^ We find a σ_abs_ value of 7.8 × 10^–21^ cm^2^ at 980 nm, which perfectly matches the result of Figure 5a. This demonstrates that the methods presented here provide a reliable σ_abs_. Together with existing methods to determine η_PL_ and *S*_r_, it should now be possible to predict the uncertainty of temperature measurements for any particular experimental setting. This shows how valuable it is to consider absorption cross sections and the quantum yields in the design of future and existing thermometers.

In conclusion, we have characterized how experimental conditions affect the uncertainty of temperature measurements through (nano)thermometry based on luminescence intensity ratios. We first measured all noise sources associated with photon detection and developed statistical models to quantitatively predict the temperature uncertainty in a wide range of temperatures and for various experimental settings. We observed that enhancement of the luminescence signal by applying EM gain significantly reduces the uncertainty until readout noise is overcome. In addition, we studied the impact of background emissions, which is a realistic practical issue. Background increases the uncertainty of a temperature measurement even if it is properly subtracted from the measurement. Our work demonstrates that the temperature uncertainty is not an intrinsic property of a luminescent (nano)thermometer but instead strongly depends on the photodetector and measurement conditions. We propose a guideline of how to compare different thermometers in a way that is relevant irrespective of the spectroscopic equipment used or of the sample under consideration. Such new ways of comparing luminescent (nano)thermometers are essential to develop and choose the ideal thermometer for the desired application.

## Supporting Information Available

Experimental procedure and additional results; experimental uncertainties at high excitation intensities; analytical expressions for the probability distributions of electron multiplication; and simulations of the uncertainties introduced by electron multiplication (PDF)
