# Supplementary material for: Impact of Noise and Background on Measurement Uncertainties in Luminescence Thermometry
Source: ACS Photonics. 2022 Mar 11;9(4):1366–74. doi: 10.1021/acsphotonics.2c00039 (PMC9026254; doi:10.1021/acsphotonics.2c00039)
Supplement: Supplementary file 1 — ph2c00039_si_001.pdf [file ph2c00039_si_001.pdf]

Supporting information for

The impact of noise and background on measurement uncertainties in luminescence thermometry

Thomas P. van Swieten[1], Andries Meijerink[1], Freddy T. Rabouw[1*]

[1]Debye Institute for Nanomaterials Science, Utrecht University, The Netherlands

*Author to whom correspondence should be addressed; electronic mail: f.t.rabouw@uu.nl

(8 pages including 8 figures)

Experimental procedure

$NaYF_4:Er^{3+}$(2%),Yb^{3+}(18%) and $NaYF_4:Yb^{3+}$(18%) nanocrystals with a spherical shape and a diameter of roughly 30 nm were synthesized using the method of Geitenbeek *et al.*[1] The nanocrystals were dispersed in cyclohexane. A droplet of the dispersion of upconversion nanocrystals was dried on a cover slip and this was attached to a microscopy slide for mechanical strength. For the measurements at elevated temperatures a droplet of dispersion was dried directly on a microscopy slide. For the absorption measurements, the dispersion of $NaYF_4:Yb^{3+}$(18%) nanocrystals was further purified with one additional washing step. Residual NaF from the synthesis was removed by sedimentation without the addition of anti-solvent.

Acquisition of the luminescence was performed on a Nikon Ti-U inverted microscope, on which the sample was placed. The microscope contains two levels of filter cube wheels. A 50/50 beam splitter was placed in both the upper and the lower level to reflect 980 nm excitation light (Coherent OBIS LX, 150 mW) and white lamp light (Halogen Lamp 12V–100W), respectively. The excitation laser was used at full power to minimize laser instabilities. The excitation light passed though neutral density filters with an optical density of 4. These light sources were directed to the sample via an air objective (Nikon CFI S Plan Fluor ELWD 60XC) to focus the excitation light on the sample. The upconversion luminescence and reflection of the lamp light was then guided through both beam splitters and a 775 nm shortpass filter (Edmund Optics) towards a spectrograph (Andor Kymera 193i) equipped with an EMCCD detector (Andor iXon Ultra 888). The measurements in conventional CCD and EMCCD modus were acquired with a readout rate of 1 MHZ and 30 MHz, respectively. The spectral acquisition times were 1000 ms and 500 ms for the CCD and EMCCD measurements, respectively. The pre-amplifier gain was set to 2 for all measurements. A heating stage (Linkam THMS600) was used to perform the measurements at elevated temperatures. The microscopy slide was pressed against the heating element to ensure good thermal contact. The front window of the stage was replaced by a home-made piece of glassware that enables a working distance of down to 2 mm. A continuous flow of nitrogen gas was directed through the stage.

Supporting discussion

To predict the uncertainty of thermometers based on a spectral shift $\Delta\lambda$ we consider an emission band with a Gaussian shape, which has mean value $\bar{\lambda}$ and width δ. A recorded photon constitutes a measurement of the emission wavelength $\bar{\lambda}$ with an uncertainty of δ. If we use $\bar{n}$ photons to estimate $\bar{\lambda}$, we achieve an uncertainty of the peak position of $\delta/\sqrt{\bar{n}}$. The uncertainty of a measurement based on the spectral shift thus depends on both the spectral width and the signal strength. Similar to Equation 3 in the main text, we can write the temperature uncertainty of these measurements as

$$\sigma_T = \sqrt{\left(\frac{\partial T}{\partial \bar{\lambda}}\right)^2 \sigma_{\bar{\lambda}}{}^2} = \frac{1}{S_\mathrm{r}} \frac{\delta}{\bar{\lambda}\sqrt{\bar{n}}}$$

where

$$S_\mathrm{r} = \frac{1}{\bar{\lambda}} \left| \frac{\partial \bar{\lambda}}{\partial T} \right|$$

This allows us to compare the temperature uncertainty as a function of signal strength for measurements based on intensity ratio and spectral shift (Figure S1). We observe that the temperature uncertainties of both methods show the same scaling with signal strength—the only difference is the absolute value. In practice, experimental factors such as the spectral resolution increase the absolute uncertainty of spectral-shift thermometers. The above equations thus only give qualitative insight in the relation between spectral width, signal strength, and the temperature uncertainty.

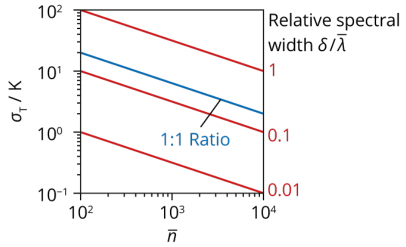

Figure S1. Calculated temperature uncertainty for intensity-ratio (blue line) and spectral-shift thermometers (red lines) with relative sensitivities of 1% K^{-1} and 0.1% K^{-1}, respectively. In both cases, $\bar{n}$ is the total counts within the entire spectrum. The uncertainty of the ratiometric thermometer is calculated with a 1:1 intensity ratio and assuming Poissonian counting noise. Different values of the relative spectral width $\delta/\bar{\lambda}$ were used to demonstrate the effect of this parameter on the temperature uncertainty."

Supporting equations

We used the work of Harpsøe *et al.* to calculate the distribution of output electrons after the electron multiplication registers of our EMCCD camera[2]

$$p_{\mathrm{EM}}\left(\bar{k}, n_{\mathrm{out}}\right)=\left((1-m\, p_{\mathrm{s}})\, \mathcal{N}(n_{\mathrm{out}}, \sigma_{\mathrm{r}})+p_{\mathrm{s}} \sum_{l=1}^{m} p_{\mathrm{gamma}}\left(n_{\mathrm{out}}, 1, G^{(m-l)/m}\right)\right) p_{\mathrm{poisson}}\left(0, \bar{k}\right) + \sum_{k=1}^{\infty} p_{\mathrm{gamma}}(n_{\mathrm{out}}, k, G)\, p_{\mathrm{poisson}}\left(k, \bar{k}\right) \tag{S1}$$

Here, the first term describes readout noise with a Gaussian distribution $\mathcal{N}(\bar{n}_{\mathrm{oe}}, \sigma_{\mathrm{r}})$ and noise due to spurious electrons, which are the unwanted electrons created during shifting of charges through the multiplication register with an overall probability p_{s} per readout. This process can occur in any of the m multiplication registers ($m = 604$ for our camera). After creation of such an electron in the lth register, it is amplified $m-l$ times. Equation S1 approximates that readout and spurious noise are relevant only if zero photoelectrons enter the multiplication register [which has a probability $p_{\mathrm{poisson}}\left(0, \bar{k}\right)$], because amplified photoelectrons otherwise dominate the probability distribution p_{EM}. The second term calculates the distribution of output electrons after multiplication of at least one photoelectron. The probability of obtaining the specific number of photoelectrons k is given by the Poisson distribution.

We derive the expected value ($\bar{n}_{\mathrm{out}}$) and the variance (σ_n) of the multiplied photoelectrons as follows

$$\bar{n}_{\mathrm{out}}=\int_{0}^{\infty} n_{\mathrm{out}} \sum_{k=1}^{\infty} p_{\mathrm{gamma}}(n_{\mathrm{out}}, k, G)\, p_{\mathrm{poisson}}\left(k, \bar{k}\right) \mathrm{d}n=\bar{k} G \tag{S2}$$

$$\sigma_{n}=\int_{0}^{\infty}\left(n_{\mathrm{out}}-\bar{k} G\right)^{2} \sum_{k=1}^{\infty} p_{\mathrm{gamma}}(n_{\mathrm{out}}, k, G)\, p_{\mathrm{poisson}}\left(k, \bar{k}\right) \mathrm{d}n=2 \bar{k} G^{2}-\bar{k}^{2} G^{2} \mathrm{e}^{-\bar{k}} \tag{S3}$$

When $\bar{k}$ is much smaller or much larger than one, the second term of the variance is negligible and the expression simplifies to $2\bar{k}G^2$. For $\bar{k}$ values around 1, this simplification no longer holds, leading to a deviation from the full expression of at most 23%. The experimental uncertainties in Figure 3b are therefore determined with $\bar{k}$ values that are much larger than one.

Supporting figures

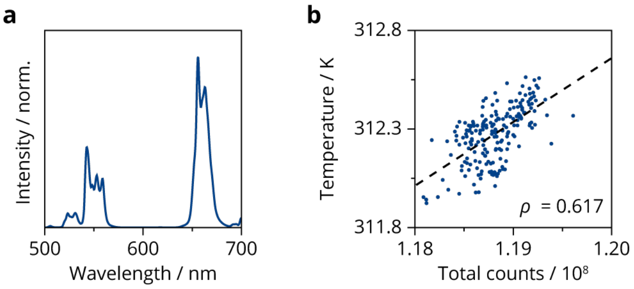

Figure S2. (a) Upconversion luminescence of dried $NaYF_4$:Er^{3+}(2%),Yb^{3+}(18%) nanocrystals upon 980-nm excitation. The excitation laser was used at full power without neutral density filters in the optical path. The laser was focused on the back focal plane of the objective to achieve wide-field excitation. (b) Correlation between the measured temperature and the total counts within 516–534 nm and 538–545 nm for the $^{2}H_{11/2}$ and $^{4}S_{3/2}$ emission, respectively. The correlation coefficient (ρ) of the linear regression is much larger than zero, indicating that measured temperature and total counts are correlated. This is likely due to fluctuations in the intensity of the excitation laser, which cause fluctuations in laser-induced heating of the nanocrystals. In our experiment of the main text, we prevented such effects by setting the illumination intensity four orders of magnitude lower.

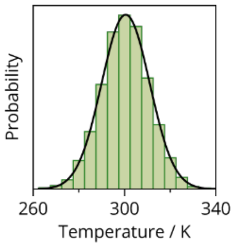

Figure S3. Histogram of temperatures extracted from 10^4 simulated spectra constituting two emission lines. The expected number of photons in a spectrum was assumed to follow a Gaussian distribution with a standard deviation of 25% reflecting fluctuations in excitation intensity. The average expected number of photons was set to $\langle\overline{n_\mathrm{A}}\rangle + \langle\overline{n_\mathrm{B}}\rangle$ = 500 counts and Poissonian noise was added to each spectrum. The solid line corresponds to the temperature distribution that is calculated using Equation S3 of the main text by simply replacing the expected photon counts $\overline{n_\mathrm{A,B}}$ with the averaged expected photon counts $\langle\overline{n_\mathrm{A,B}}\rangle$.

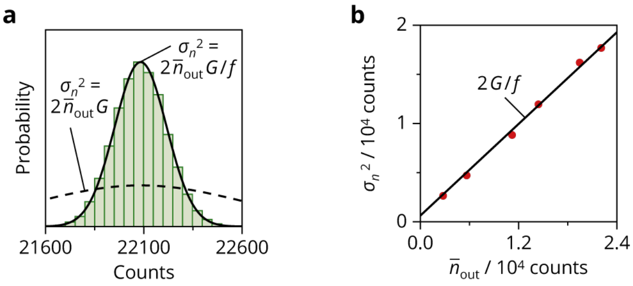

Figure S4. (a) The distribution of counts per 100-ms frame, for pixels on our EMCCD camera showing an average of 22085 counts / 100 ms when measured over 200 frames. The camera recorded the reflection of a white lamp

on a microscopy slide at an EM gain level of 2. The solid line is a fit of the experimental data to the normal distribution ($\bar{k} = 22085$, $\sigma^2 = 17690$) and the dashed line shows the distribution of output electrons according to Equations S2–S3 with $\bar{k} = 22085$. (b) Plot of the variance against the mean (red dots) measured via the procedure in Figure (a). The black line is a fit of the experimental data to the model ${\sigma_n}^2 = 2\bar{n}_{\mathrm{out}}G/f + {\sigma_{\mathrm{r}}}^2$, where $f = 5.14$ is the analog-to-digital conversion factor and ${\sigma_{\mathrm{r}}}^2 = 630$ is the readout variance of one pixel.

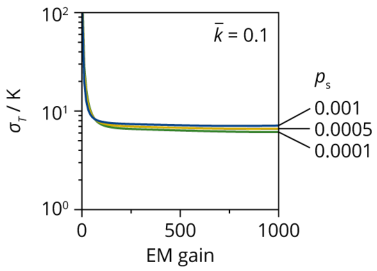

Figure S5. Temperature uncertainty for a range of total EM gains and various probabilities of generating spurious electrons p_s, covering the range that we expect to encounter in our EMCCD. We estimate that these probabilities are 2 to 3 orders of magnitude lower than the probability of multiplying a real photoelectron at an EM gain of 1000. The uncertainties are calculated from the expected value of the output electrons, excluding spurious electrons, and from the variance of all output electrons. We use an extremely low value of expected photoelectrons $\bar{k} = 0.1$ as input and observe only a very weak effect of spurious charges on the temperature uncertainty. This indicates that for more realistic values of $\bar{k}$ the impact of spurious charges on the temperature uncertainty is negligible.

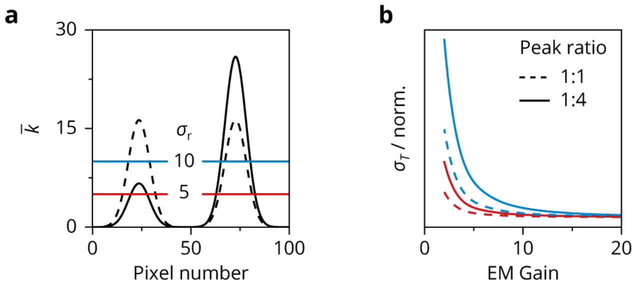

Figure S6. (a) Two bands with Gaussian shape and a peak ratio of 1:4 (solid black line) and 1:1 (dashed black line). The integrated area below the dashed and solid black lines is equal. The red and blue line correspond to readout noise levels of 5 and 10, respectively. (b) Calculated temperature uncertainty for the different scenarios in (a). The blue curves show the uncertainty for a readout noise level of 10 and a peak ratio of 1:4 (solid line) and 1:1 (dashed line). Same for the red curves but for a readout noise level of 5. The uncertainties are calculated from the single-pixel expected values of output electrons, excluding spurious electrons, and from the single-pixel variances of all output electrons, followed by summing the values for the individual pixels within each emission band. Each curve is individually normalized to its minimum uncertainty, which in each case occurs at the highest EM gain.

Figure S6 considers the effect of the significantly differing emission intensities in the two emission bands. Of particular interest is the scenario in which the signal in one emission band is weaker than the readout noise, while the other band exceeds the readout noise. To understand the impact on the

temperature uncertainty we simulate two emission bands as Gaussians (Figure S6a). We then calculate the temperature uncertainty of these scenarios using Equation 3 and 7 of the main text (Figure S6b). EM gain has a stronger beneficial effect on the temperature uncertainty if the emission spectrum is asymmetric (solid lines) and if the readout noise is higher (blue lines).

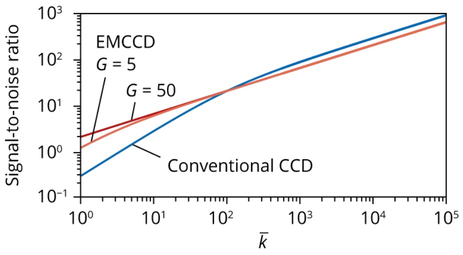

Figure S7. Signal-to-noise ratio for a conventional CCD and an EMCCD with a multiplication gain G of 5 and 50. In all cases, the read-out variance $\sigma_r{}^2$ was set to 100 and the ADC factor was set to 1. Compared to the EMCCD, the signal-to-noise ratio of a conventional CCD is poor at a low number of expected photoelectrons $\bar{k}$, but it outperforms the EMCCD above 100 photoelectrons.

Characterization of the absorption cross section

The absorption spectrum was acquired on a double-beam PerkinElmer Lambda 950 UV/vis/NIR spectrometer. After the measurement, the dispersion was dried to determine the nanocrystal concentration. Thermogravimetric analysis (Q50, TA Instruments) was performed to determine the mass fraction of the ligands (4.4%), which was subtracted from the dried mass to obtain the $NaYF_4$:Yb^{3+}(18%) concentration. A home-built setup, shown in Supporting Figure 8a, was used to determine the absorption cross section from the saturation characteristics of $NaYF_4$:Yb^{3+}. Microcrystalline $NaYF_4$:Yb^{3+}(18%) was prepared via the procedure in Krämer *et al.*[3] A thin layer of powder was attached to a microscopy slide using double-sided scotch tape (Figure S8b). To control the excitation intensity on the sample, the light output from a continuous wave 2W 980 nm laser (MDL-III) was directed through neutral-density filters and a lens. A 1000 nm shortpass filter (Thorlabs FESH1000) was used to clean the laser spectrum. Two lenses were used to guide the emission to a fiber that was connected to a spectrometer (Andor Kymera 193i) equipped with a water-cooled CCD detector (Andor iDus 1.7µm InGaAs). A 1000 nm longpass filter (Thorlabs FESH1000) was used to reject the excitation light. The excitation intensity at the sample was determined by measuring the output power of the laser (2.16 W) and characterizing the spot size with a simple CCD camera (Thorlabs DCU223C) (Figure S8c).

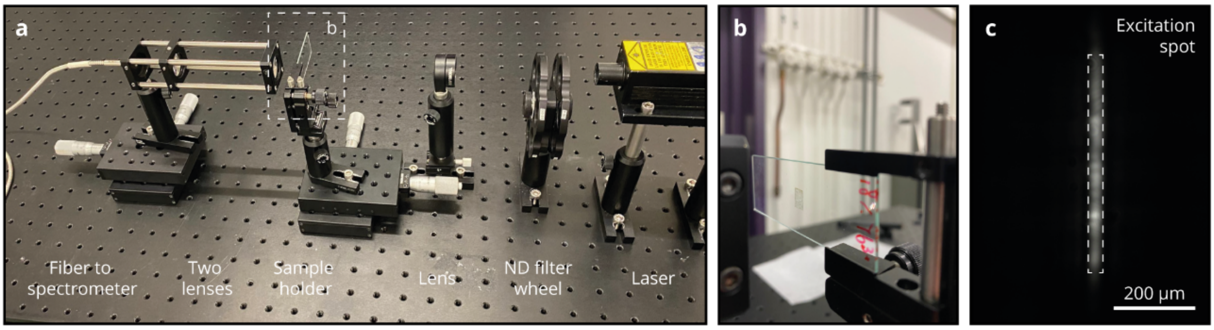

Figure S8. Luminescence saturation setup. (a) Photograph of the setup. (b) Zoom-in of the microcrystalline $NaYF_4$:Yb^{3+}(18%) sample attached to microscopy slide in (b) using double-sided scotch tape. (c) Image of the 980 nm excitation spot on the glass slide near the sample. The divergence of the laser was larger in one direction, which resulted in an elongated shape of the focused laser spot. The white dashed line indicates the spot size from which the excitation intensity was determined.

Supporting references

1 Geitenbeek, R. G.; Prins, P. T.; Albrecht, W.; Van Blaaderen, A.; Weckhuysen, B.M.; Meijerink, A. $NaYF_4$:Er^{3+},Yb^{3+}/SiO_2 Core/Shell Upconverting Nanocrystals for Luminescence Thermometry up to 900 K. *J. Phys. Chem. C* **2017**, *121*, 3503–3510.

2 Harpsøe, K. B. W.; Andersen, M. I.; Kjægaard, P. Bayesian Photon Counting with Electron-Multiplying Charge Coupled Devices (EMCCDs) *Astron. Astrophys.* **2012**, *537*, A50.

3 Krämer, K. W.; Biner, D.; Frei, G.; Güdel, H. U.; Hehlen, M. P.; Lüthi, S. R. Hexagonal Sodium Yttrium Fluoride Based Green and Blue Emitting Upconversion Phosphors. *Chem. Mater.*, **2004**, *16*, 1244–1251.
